# Predicting orthodontic appointment adherence in adolescents and parents using an extended behavioral model

**DOI:** 10.1093/ejo/cjag065

**Published:** 2026-10-06

**Authors:** Maryam Elyasi, Babak Bohlouli, Akriti Tiwari, Maryam Amin

**Affiliations:** Mike Petryk School of Dentistry, University of Alberta, 5-513 Dianne and Irving Kipnes Health Research Academy, Edmonton, Alberta, T6G 1C9, Canada; Mike Petryk School of Dentistry, University of Alberta, 5-513 Dianne and Irving Kipnes Health Research Academy, Edmonton, Alberta, T6G 1C9, Canada; Mike Petryk School of Dentistry, University of Alberta, 5-513 Dianne and Irving Kipnes Health Research Academy, Edmonton, Alberta, T6G 1C9, Canada; Mike Petryk School of Dentistry, University of Alberta, 5-513 Dianne and Irving Kipnes Health Research Academy, Edmonton, Alberta, T6G 1C9, Canada

**Keywords:** orthodontics appointment adherence, adolescents, theory of planned behavior, sense of coherence, structural equation modeling

## Abstract

**Background:**

Poor adherence in orthodontic care is a complex issue involving both children and their parents. The study aimed to expand the theory of planned behavior (TPB) by integrating sense of coherence (SOC) to predict appointment attendance in adolescent orthodontic patients.

**Materials and methods:**

This prospective cohort study recruited adolescents from the University of Alberta Graduate Orthodontic Clinic. Baseline questionnaires captured sociodemographic data, TPB constructs, and SOC. Appointment attendance and oral hygiene were monitored over 1 year. Structural equation modeling (SEM) was used to examine relationships between TPB constructs and appointment adherence, with model fit assessed using χ^2^, chi-square/degrees of freedom ratio (CMIN/DF), root mean square error of approximation (RMSEA), and comparative fit index (CFI).

**Results:**

A total of 168 adolescent–parent pairs participated, and 57% of participants missed at least one appointment during the first year of treatment. SEM demonstrated moderate model fit (CMIN/DF = 1.74; RMSEA = 0.06; CFI = 0.84). Within the TPB constructs, only perceived behavioral control (PBC) significantly predicted intention (*P* < 0.05), whereas attitude and subjective norm were not significant predictors. SOC did not significantly predict intention, TPB constructs, or appointment adherence in either the adolescent or parent models. Intention also failed to significantly predict actual attendance.

**Conclusion:**

Within the expanded TPB–SOC framework, PBC was the sole psychosocial predictor of orthodontic appointment-adherence intention. However, neither intention nor SOC translates into actual clinical attendance. The moderate model fit indicates that psychosocial constructs alone are insufficient to fully explain orthodontic appointment adherence. To build more effective adherence models, future research and clinical strategies must look beyond psychology and account for structural, logistical, and environmental barriers.

## Introduction

Lack of adherence to professional health care advice among adolescents is a major health concern and a complex issue, as it involves the roles of both the adolescent and their parents [1]. Regardless of how effective preventive or therapeutic measures may be, the quality of care is undermined if parents and adolescents fail to follow recommendations provided by their healthcare professionals [2]. Orthodontic treatment in adolescents provides an ideal context for assessing adherence to oral health care because of its long duration, typically lasting 2–3 years [3]. Since orthodontic care primarily occurs in outpatient settings, it requires ongoing self-care behaviors from patients alongside continuous parental involvement [4]. Lack of adherence during active treatment often prolongs treatment duration and increases plaque retention, thereby raising the likelihood of negative outcomes such as white spot lesions (WSLs) [5].

In a study examining adherence behaviors among orthodontic patients, orthodontists rated “the patient keeps appointments” as the most important factor [6]. Regular dental visits have also been shown to significantly improve adolescents’ oral health, with both personal attitudes and social norms positively influencing adherence to appointment attendance [7]. Importantly, parents’ perceptions and attitudes toward dental visits for adolescents strongly determine adherence to scheduled appointments. Negative experiences such as an unpleasant first visit, dissatisfaction with prior care, uncertainty about treatments, or adolescents’ fear and resistance to dental care can discourage parents from ensuring regular attendance, thereby influencing adolescents’ adherence behavior to orthodontic treatment [8]. In addition, appliance maintenance requires considerable commitment and reflects patients’ adherence behavior; however, research suggests that more than half of patients experience difficulties maintaining their orthodontic appliances [9]. Of the three major adherence-related behaviors, oral hygiene, appliance care, and appointment attendance are influenced by both adolescents and their parents, although parents bear greater responsibility as they arrange and facilitate visits [9].

Theories have proven to be valuable frameworks for oral health research. One such theory, the theory of planned behavior (TPB), has demonstrated its effectiveness in explaining a significant proportion of the variance in oral health behaviors among adults [10]. However, its application to adolescents’ and parents’ adherence behavior in orthodontic treatment remains relatively new. Although some studies have applied TPB to assess adherence behavior in adolescents and/or adults undergoing orthodontic treatment [11] and to examine intentions to improve oral health behaviors [12], to date, no research has specifically explored orthodontic adherence among adolescents and their parents through the lens of TPB. A notable strength of TPB lies in its flexibility to incorporate additional constructs relevant to specific behaviors, allowing integration with other adherence-related variables [10].

Coping with daily stressors represents an essential psychological resource that supports individuals in maintaining healthy practices. Among such variables, the ability to cope effectively plays an essential role in identifying and mobilizing resources for sustaining healthy behaviors [13]. This ability is reflected in the concept of sense of coherence (SOC), the central element of the salutogenic model, which explains how individuals manage stress and maintain health. As Antonovsky emphasized, individuals with a strong SOC perceive life as structured, manageable, and meaningful and are therefore more capable of recognizing and utilizing resources to meet demands. Research has consistently linked SOC with stronger intentions to adhere to preventive and therapeutic health measures [14].

Existing research on adherence behavior in orthodontics underscores the crucial role of coping skills for both patients and their parents [4, 15]. For patients, the main challenges include coping with pain and discomfort, as well as maintaining oral health practices, which are often cited as reasons for treatment discontinuation. For parents, their ability to encourage and support their children in adhering to treatment recommendations and oral care guidelines from healthcare providers plays a vital role in determining treatment outcomes. Notably, children's cooperation during treatment tends to increase when they perceive strong parental support [16, 17].

The objective of this study was to develop an expanded theoretical model that integrates the concept of SOC into the original TPB framework to predict appointment attendance among orthodontic patients aged 12–18 years. The development of this expanded model can help identify the relevant variables to be analyzed, anticipate potential interactions between them, and ultimately optimize treatment outcomes.

## Materials and methods

### Adherence definition and assessment

Adherence in orthodontics refers to the extent to which patients follow recommendations necessary for successful treatment outcomes, including attendance at scheduled appointments, maintenance of orthodontic appliances, retainer wear, and adherence to oral-hygiene instructions [9, 18]. Among these behaviors, appointment attendance has consistently been identified by orthodontists as one of the most important indicators of treatment adherence [19]. In the present study, appointment adherence was assessed prospectively during the first year of treatment. Patients’ adherence to the scheduled appointment was recorded as appointment attendance during the 1-year period. The appointment was considered as “failed/missed” if the patient did not show up, canceled the appointment on the same day, or came too late causing rescheduling [20]. Although these behaviors may arise from different underlying causes, all contribute to disruption of clinic scheduling, inefficient use of chair time, and potential prolongation of treatment duration [21].

### Study design and setting

This prospective, longitudinal, single-center study was conducted at the University of Alberta (UofA) Graduate Orthodontic Clinic. Ethical approval was obtained from the Institutional Review Board (name and approval number blinded for peer review).

### Participants

This clinic-based cohort study was carried out from August 2017 to November 2019. A representative consecutive sample of orthodontic adolescent patients aged 12–18 years who were scheduled to begin maxillary and mandibular fixed multibracket orthodontic therapy, along with their parents, was recruited from the orthodontic department using a convenience sampling method. Participants were excluded if they had a chronic systemic disease or significant medical history, craniofacial developmental anomalies, including cleft lip and palate, hypodontia, previous orthodontic treatment, difficulty reading or speaking English, or if they declined participation. A calibrated research assistant and an orthodontic graduate student explained the study to the participants and their legal guardians and obtained written informed consent from those who agreed to participate.

### Sample size calculation

Based on a study of adherence behaviors among orthodontic patients, “The patient keeps appointments” was the highest rated factor regarding the importance from orthodontists’ perspective [19]. Therefore, considering the 11.6% of appointment nonattendance, as one of the adherence behaviors for orthodontic treatments, at the UofA orthodontic graduate program and based on 5% standard error, 95% confidence interval, the estimated sample size was 140 orthodontic patients and their parents. An additional 20% of the calculated sample size was added to the minimum sample size to compensate for possible losses. Therefore, a total of 168 child orthodontic patients and their parents were recruited.

### Data collection and measures

Data were collected prospectively using self-administered questionnaires and clinical records. Baseline questionnaires were completed prior to treatment initiation and required ∼20 minutes. Sociodemographic data included adolescent age and gender and parent age, relationship to the adolescent, education level, and household income. Adolescent and parent attitudes, subjective norms (SBJN), perceived behavioral control (PBC), and intention regarding orthodontic appointment adherence were assessed using TPB-based questionnaires. Intention was measured using a single item. Responses were recorded on 7-point Likert scales (“strongly disagree” to “strongly agree”). SOC was assessed using the 13-item short-form SOC questionnaire (SOC-13; score range 13–91).

Oral-hygiene adherence was evaluated by visual examination of labial and buccal WSLs using a standardized 0–3 grading scale (Table 1) after 5 seconds of air drying at appliance placement (T0) and at 4–6 months (T1), and 12 months (T3). In addition, adolescents self-reported their daily toothbrushing frequency. Appointment adherence was recorded prospectively over one year using electronic scheduling records. Missed appointments were defined as nonattendance, same-day cancellation, or late arrival requiring rescheduling.

**Table 1:** WSL measurements.

Grade	Presentation
0	No visible WSL or surface disruption (no demineralization)
1	Visible WSL without enamel surface disruption (mild demineralization)
2	Visible WSL with roughened enamel surface (moderate demineralization)
3	Visible WSL requiring restoration (severe demineralization)

### Statistical analysis

Descriptive statistics were used to summarize participant characteristics, including means and standard deviations for continuous variables and frequencies and percentages for categorical variables. Internal consistency of questionnaire scales was assessed using Cronbach's alpha coefficients. Structural equation modeling (SEM) was conducted using IBM SPSS AMOS to examine relationships among TPB constructs and intention to attend orthodontic appointments. Model fit was evaluated using the chi-square test (χ^2^), the chi-square/degrees of freedom ratio (CMIN/DF), the root mean square error of approximation (RMSEA), and the comparative fit index (CFI). Model fit was considered acceptable based on commonly used criteria (CMIN/DF <2.0, RMSEA ≤0.08, and CFI ≥0.80). Statistical significance was set at *P* < 0.05.

## Results

The study achieved a response rate of 98%, with no missing data or loss to follow-up. A total of 168 adolescent–parent pairs participated. Adolescents had a mean age of 14 ± 1.5 years, and parents had a mean age of 44 ± 5 years. Among adolescents, 54% were female, and 37% had siblings who were receiving or had previously received orthodontic treatment. Most parents were mothers (80.4%), 43% of families reported a monthly income ≥$5000, and 80% of parents had attained postsecondary or college education. Participant demographic and socioeconomic characteristics are summarized in Table 2.

**Table 2:** Participants characteristics (*N* = 168).

Age	Mean (standard deviation)
Child	14 (1.5)
Parent	44 (5.1)
Gender	*N* (%)
Male	78 (46)
Female	90 (54)
Sibling with orthodontic treatment, *N* (%)	63 (37)
Mother education	*N* (%)
Secondary school or less	35 (21)
Some postsecondary or college	73 (43)
Postsecondary	60 (37)
Monthly household income	*N* (%)
$2000–$2999	11 (7)
$3000–$4999	85 (50)
Over $5000	72 (43)

Clinical assessment revealed a progressive increase in labial and buccal WSLs over the first year of orthodontic treatment. At baseline (T0), 22% of adolescents presented with at least one WSL, all classified as mild (grade 1). This proportion increased to 48% at T1 (4–6 months), with 79% mild (Grade 1) and 21% moderate (Grade 2) lesions, and further to 59% at T2 (12 months), comprising 53% mild, 39% moderate, and ∼9% severe (Grade 3) lesions (Table 3). At T0, self-reported oral-hygiene behaviors were consistent between parents and adolescents, with 76% of parents and 73% of adolescents reporting tooth brushing at least twice daily. Appointment adherence during the first year was varied: 43% of adolescents attended all scheduled visits, 32% missed at least one appointment, 11% missed two appointments, and 14% missed three or more appointments (Table 3).

**Table 3:** Adherence-related characteristics of orthodontic child patients (*N* = 168).

Adolescents’ toothbrushing frequency (adolescents’ response)	<2×/day		46 (27%)	
	≥2×/day		122 (73%)	
Adolescents’ toothbrushing frequency (parent's response)	<2×/day		44 (24%)	
	≥2×/day		124 (76%)	
Patients with labial/buccal WSL(s)^a^	T0		37 (22%)	
	T1		81 (48%)	
	T2		97 (59%)	
Severity of labial/buccal WSL(s)		G_1_^b^	G_2_^c^	G_3_^d^
	T0	37 (100%)	NA	NA
	T1	65 (79%)	15 (21%)	NA
	T2	57 (53%)	32 (39%)	8 (9%)
Missed appointments	0		78(43%)	
	1		55 (32%)	
	2		17 (11%)	
	3 or more		18 (14%)	

NA, not available.

^a^White spot lesion.

^b^Grade_1_: visible WSL without enamel surface disruption (mild demineralization).

^c^Grade_2_: visible WSL with roughened enamel surface (moderate demineralization).

^d^Grade_3_: visible WSL requiring restoration (severe demineralization).

A subset of items was used to assess TPB constructs. Attitude was measured with eight 5-point Likert-type items (Cronbach's α = 0.74), SBJN with nine 5-point items (α = 0.82), and PBC with five 5-point items (α = 0.80). SOC was assessed using thirteen 7-point items (α = 0.87). Internal consistency coefficients above 0.70 are generally considered acceptable. Considering the sensitivity of Cronbach's alpha to a low number of items, the average alpha exceeded 0.90, indicating homogeneity across scales (Table 4).

**Table 4:** Item-total statistics.

	Scale mean if item deleted	Scale variance if item deleted	Corrected item-total correlation	Squared multiple correlation	Cronbach’s alpha if item deleted
1. R_SOC	124.450	194.632	0.368	0.309	0.904
2. R_SOC	124.670	193.802	0.364	0.544	0.904
3. R_SOC	124.640	190.784	0.476	0.592	0.902
4_SOC	126.410	195.226	0.382	0.297	0.903
5_SOC	126.180	191.253	0.513	0.436	0.901
6_SOC	126.370	189.037	0.583	0.424	0.900
7. R_SOC	124.390	190.610	0.587	0.599	0.900
8_SOC	126.540	187.999	0.497	0.556	0.901
9_SOC	126.420	186.891	0.545	0.623	0.900
10.R_SOC	124.450	192.668	0.409	0.355	0.903
11_SOC	126.730	191.742	0.450	0.481	0.902
12_SOC	126.330	190.116	0.497	0.434	0.901
13_SOC	126.300	187.805	0.563	0.573	0.900
Gender	129.740	207.967	−0.171	0.237	0.909
1_Attitude	125.730	197.194	0.488	0.702	0.902
2_Attitude	125.770	195.182	0.548	0.729	0.901
3_Attitude	125.800	195.863	0.484	0.702	0.902
4. R_Attitude	124.390	190.095	0.480	0.460	0.902
5_R_Attitude	124.120	190.824	0.574	0.483	0.900
6_SBIN	126.110	194.269	0.475	0.585	0.902
7_SBJN	126.660	193.687	0.391	0.455	0.903
8_SBJN	126.450	192.920	0.511	0.492	0.901
9_SBJN	126.010	195.527	0.465	0.686	0.902
10_SBJN	125.740	195.473	0.583	0.716	0.901
11_PBC	126.020	191.347	0.546	0.643	0.900
12. R_PBC	124.280	187.999	0.558	0.526	0.900
13_PBC	125.980	192.664	0.620	0.613	0.900
14. R_PBC	124.320	189.166	0.551	0.546	0.900
15. R_PBC	124.670	188.667	0.451	0.466	0.903

SEM demonstrated acceptable fit (χ^2^ = 883.12, df = 507, *P* < 0.001; CMIN/DF = 1.74; RMSEA = 0.06; CFI = 0.84). SEM results revealed that PBC significantly predicted intention (*P* < 0.05), whereas attitude, SBJN, intention did not significantly contribute to the model. Predisposing factors, including age, gender, and having a sibling with previous orthodontic treatment, had no significant impact (*P* > 0.05). Additionally, SOC did not significantly influence attitude, PBC, or SBJN (*P* > 0.05). The measurement and structural components of the model are illustrated in Fig. 1, and the results are summarized in Table 5 (Supplementary material I presents the model).

**Figure 1:**
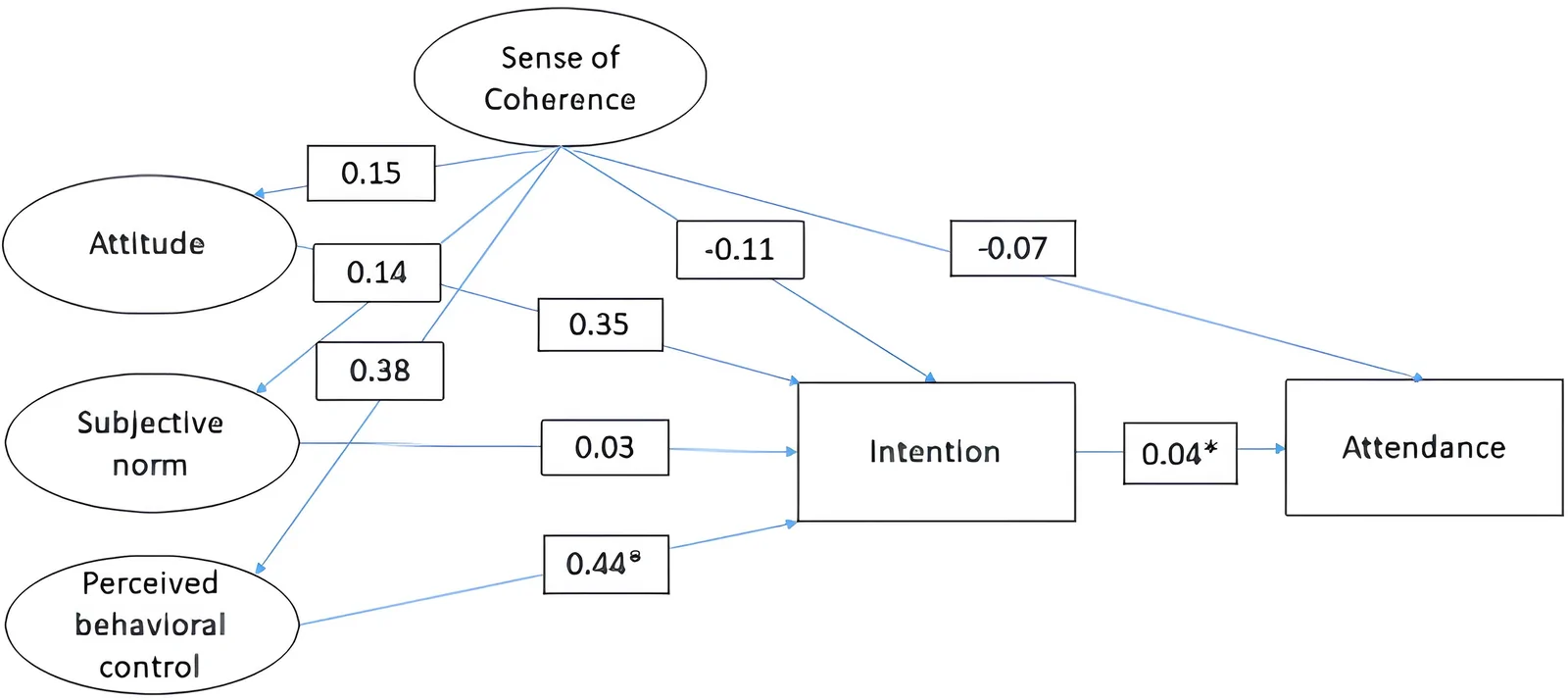
Causal model path β coefficients, *P < 0.05.

**Table 5:** Regression results for children's impact on attendance model.

			Estimate	S.E.	C.R.	*P*
Attitude	←	SOC	1			
Attitude	←	Age	0.00	0.00	0.21	0.84
Attitude	←	Ortho_other_Child	0.01	0.04	0.21	0.84
SOC_3_R	←	SOC	103.65	503.23	0.21	0.84
SBJN	←	SOC	31.81	154.57	0.21	0.84
PBC	←	SOC	64.73	314.09	0.21	0.84
SBJN	←	Age	0.01	0.01	0.92	0.36
PBC	←	Age	0.04	0.01	3.92	^***^
SBJN	←	Ortho_other_Child	0.06	0.06	0.98	0.33
PBC	←	Ortho_other_Child	0.20	0.11	1.88	0.06
PBC	←	Attitude	21.19	102.93	0.21	0.84
SBJN	←	Attitude	13.14	63.68	0.21	0.84
Attitude_5_R	←	Attitude	1.00			
Intention	←	Attitude	15.44	74.72	0.21	0.84
Intention	←	PBC	0.24	0.06	3.73	^***^
Intention	←	SBJN	0.12	0.12	0.99	0.32
Attitude_5_R	←	PBC	0.62	0.10	6.35	^***^
SOC_2_R	←	SOC	16.54	81.95	0.20	0.84
Intention	←	SOC	10.20	50.40	0.20	0.84
SOC_2_R	←	SOC_3_R	0.59	0.06	10.39	^***^
Intention	←	Age	−0.01	0.01	−1.86	0.06
Attitude_4	←	Attitude	37.79	182.97	0.21	0.84
Attitude_3	←	Attitude	35.97	174.17	0.21	0.84
Attitude_2	←	Attitude	39.13	189.49	0.21	0.84
Attitude_1	←	Attitude	38.31	185.51	0.21	0.84
PBC_1	←	PBC	1.00			
PBC_2	←	PBC	0.64	0.05	11.87	^***^
PBC_3_R	←	PBC	0.86	0.09	9.70	^***^
PBC_4	←	PBC	0.87	0.06	15.81	^***^
PBC_5_R	←	PBC	0.81	0.09	9.02	^***^
SBJN_5	←	SBJN	1.00			
SBJN_4	←	SBJN	1.38	0.14	10.18	^***^
SBJN_3	←	SBJN	1.59	0.22	7.30	^***^
SBJN_2	←	SBJN	1.69	0.22	7.54	^***^
SBJN_1	←	SBJN	1.62	0.19	8.40	^***^
Missed appoint proportion	←	Intention	0.02	0.03	0.70	0.48
Missed appoint proportion	←	Attitude	−0.72	3.62	−0.20	0.84
Missed appoint proportion	←	PBC	−0.07	0.02	−3.56	***
Missed appoint proportion	←	SBJN	−0.01	0.04	−0.20	0.84
SOC_1_R	←	SOC	1.00			
SOC_4	←	SOC	96.21	466.85	0.21	0.84
SOC_5	←	SOC	166.81	809.27	0.21	0.84
SOC_6	←	SOC	187.28	908.51	0.21	0.84
SOC_7_A	←	SOC	92.47	448.76	0.21	0.84
SOC_8	←	SOC	168.08	815.38	0.21	0.84
SOC_9	←	SOC	196.50	953.23	0.21	0.84
SOC_10_R	←	SOC	104.84	508.79	0.21	0.84
SOC_11	←	SOC	135.40	656.88	0.21	0.84
SOC_12	←	SOC	167.00	810.20	0.21	0.84
SOC_13	←	SOC	159.36	773.07	0.21	0.84
Missed appoint proportion	←	SOC	−1.67	8.71	−0.19	0.85
Missed appoint proportion	←	Ortho_other_Child	0.01	0.02	0.68	0.50
Attitude_3	←	SOC	40.62	197.29	0.21	0.84
SOC_1_R	←	Attitude_5_R	0.26	0.07	3.89	***
SOC_1_R	←	SOC_2_R	0.21	0.06	3.39	^***^

The same analyses were conducted to examine the influence of parents on the attendance model. SEM demonstrated a moderately acceptable fit (χ^2^ = 758.64, df = 441, ***P < 0.001; CMIN/DF = 1.72; RMSEA = 0.06; CFI = 0.88). No significant associations were observed between parental attitude, SBJN, or PBC and intention or missed appointments. Additionally, SOC did not predict intention or appointment adherence (see Supplementary material II).

## Discussion

The SEM demonstrated moderate fit for both adolescent and parent populations. In the adolescent model, PBC was the only TPB construct that significantly predicted behavioral intention, whereas attitude and SBJN were not significant predictors. Predisposing factors, including age, gender, and having a sibling with previous orthodontic treatment, were not significantly associated with either intention or attendance behavior. SOC also did not significantly influence attitude, SBJN, or PBC. Interestingly, PBC was directly associated with missed appointment proportion, whereas intention was not significantly associated with attendance behavior. This finding may suggest that adolescents’ perceived ability to attend scheduled appointments and overcome practical barriers influences attendance behavior beyond behavioral intention alone. This interpretation is consistent with the TPB, which proposes that PBC may exert both indirect effects through intention and direct effects on behavior, particularly when external constraints affect an individual's ability to perform the behavior [10]. In contrast, the parent model showed no significant associations between TPB constructs, SOC, intention, or attendance behavior. Collectively, these findings suggest that PBC may be a relevant target for interventions to improve orthodontic appointment adherence; however, the overall explanatory power of the TPB–SOC framework appears limited in this context.

The findings of the present study differ from those reported by Elyasi *et al.* [22], who found that an expanded TPB model incorporating parental SOC explained a substantial proportion of variance in preschool children's preventive dental attendance. Their findings highlighted the importance of parental SOC and empowerment in promoting attendance. Similarly, Amin *et al.* [23] demonstrated that TPB effectively predicted dental attendance intentions and behaviors among children from newcomer families, although it did not predict caries experience, suggesting that additional biological and environmental factors also contribute to oral health outcomes.

Beyond TPB, previous studies have consistently shown that higher SOC in both caregivers and children is associated with better oral health behaviors, such as regular toothbrushing and lower caries incidence [24, 25]. However, Mathisen *et al.* [26] reported no significant association between SOC and clinical oral status, although SOC was positively associated with self-perceived oral health, suggesting that SOC may influence perceptions and behaviors more than objective outcomes.

In contrast, the findings of this study suggest that the predictive utility of both TPB and SOC for orthodontic appointment attendance is more limited than previously assumed. PBC emerged as the most influential TPB component among adolescents, but overall cognitive and psychosocial constructs did not fully explain attendance behavior. The absence of significant associations for parental TPB constructs and SOC further suggests that structural, contextual, or health system-related factors may play a stronger role in appointment adherence than individual beliefs or coping orientation.

SOC did not significantly predict appointment adherence intention or behavior in either model. This may be explained by several factors. SOC in adolescents aged 12–18 may be less stable than in adulthood, consistent with Antonovsky's proposition that SOC develops throughout adolescence and becomes more stable in early adulthood [27]. In addition, the study population recruited from a university-based clinic likely reflects a relatively high socioeconomic status, which may have reduced variability in SOC scores [28]. Furthermore, SOC may be more strongly associated with preventive oral health behaviors, such as routine dental attendance and toothbrushing, than with adherence to scheduled therapeutic appointments [29]. This suggests that the relationship between SOC and adherence may be context dependent and may differ across oral health behaviors. Finally, orthodontic appointment attendance is influenced by both adolescents and parents, and adolescent SOC may not fully capture the psychosocial factors underlying attendance. Although parental SOC was also not significant in this study, previous research has shown it to be relevant in preventive dental attendance, suggesting context-dependent effects across oral health behaviors [22].

Although participants reported high toothbrushing adherence, clinical observations indicated a progressive decline in oral hygiene over time. This highlights the limitations of self-reported behaviors in adolescents, particularly due to social desirability and recall bias, and underscores the importance of incorporating objective clinical indicators in orthodontic care. Additionally, 32% of patients missed at least one appointment and 14.1% missed three or more in the first year, indicating notable nonadherence. While parental influence has been identified as important in previous studies, these findings suggest that other unmeasured factors such as logistical challenges, scheduling conflicts, treatment burden, or individual motivation may play a more significant role [22].

The expanded TPB–SOC model demonstrated only moderate fit in explaining orthodontic appointment adherence. Within the model, PBC was the only significant predictor of intention, while attitude, SBJN, and SOC were not significant. Overall, these findings indicate that psychosocial constructs alone provide only a partial explanation of adherence in adolescent orthodontic patients. In addition, SOC may have limited predictive value in this context due to its developmental nature in adolescence and the relatively homogeneous socioeconomic profile of the study population. Taken together, these findings suggest that although SOC has been associated with preventive oral health behaviors in previous studies, its contribution to orthodontic appointment adherence appears limited in the present context.

An additional clinically important finding was the marked increase in WSL prevalence during the first year of treatment. WSLs increased from 22% at baseline to 59% after 12 months of treatment, with approximately half of affected patients demonstrating moderate-to-severe demineralization. WSLs are a well-recognized consequence of inadequate plaque control during fixed appliance therapy and frequently develop around orthodontic brackets when oral hygiene practices are insufficient [30]. Despite relatively high levels of self-reported toothbrushing adherence, the prevalence of WSL increased substantially over time. This discrepancy suggests that self-reported oral health behaviors may not accurately reflect actual oral-hygiene performance and highlights the limitations of relying exclusively on self-report behavioral measures. Objective clinical outcomes may therefore provide a valuable complement to psychosocial measures of adherence. From a clinical perspective, these findings reinforce the importance of ongoing monitoring of oral hygiene throughout treatment and early identification of patients at increased risk of enamel demineralization. The results also suggest that behavioral intentions and self-reported adherence may not necessarily translate into clinically adequate oral-hygiene practices. Accordingly, objective measures such as WSL assessment should be considered alongside behavioral measures when evaluating adherence during orthodontic treatment.

While the present study was conducted in a Canadian orthodontic setting, the theoretical constructs examined are not specific to a single healthcare system. The TPB has been widely applied to predict health-related and adherence behaviors across diverse populations and healthcare contexts [10], while SOC has been described as a cross-cultural construct associated with health-promoting behaviors and coping resources [31]. TPB and SOC have been used internationally to explain behavioral outcomes related to oral health and healthcare utilization. Nevertheless, differences in orthodontic service delivery models may influence the generalizability of the present findings. In healthcare systems where orthodontic treatment for adolescents is publicly funded or heavily subsidized, financial barriers to treatment may be reduced; whereas, in predominantly private systems, treatment costs and insurance coverage may play a greater role in treatment access and utilization [32]. Consequently, healthcare-system characteristics should be considered when interpreting and applying the findings to other jurisdictions. The results of this study should therefore be viewed within the context of local orthodontic care pathways, reimbursement structures, and access to treatment.

Several limitations should be considered when interpreting the findings of this study. First, participants were recruited from a single university-based graduate orthodontic clinic using a convenience sampling approach, which may limit the representativeness of the sample. Most participating parents reported postsecondary education (80%) and moderate-to-high household income levels (43% >$5000/month), potentially restricting the generalizability of the findings to populations with lower socioeconomic status or to patients receiving treatment in community-based or private orthodontic settings. In addition, the exclusion of non-English-speaking families may have introduced further selection bias and reduced the representation of culturally and linguistically diverse populations. Second, participants were followed only during the first year of orthodontic treatment, whereas comprehensive orthodontic care typically extends over 2 to 3 years. As adherence behaviors may change over the course of treatment, the findings may not fully reflect long-term adherence patterns. Finally, adherence was assessed using self-administered questionnaires, which are subject to recall bias, particularly among adolescents, and may have resulted in overreporting of adherence behaviors. These limitations should be considered when interpreting the results and highlight important areas for future research.

## Conclusion

This study examined the expanded TPB–SOC model for predicting orthodontic appointment adherence in adolescent–parent pairs. PBC significantly predicted adolescents’ intention to attend orthodontic appointments. In contrast, attitude, SBJN, and SOC were not significant predictors in either adolescents or parents, and overall model fit was moderate. These findings suggest that PBC may be the most relevant target for adherence interventions in adolescent orthodontic care. However, the model's limited predictive power indicates that other factors, such as logistical constraints, scheduling difficulties, treatment burden, and environmental influences, may also play important roles. Future research should examine these factors alongside psychosocial constructs to develop more comprehensive models of adherence. The increasing prevalence of WSLs despite high self-reported brushing further highlights the value of objective clinical monitoring in orthodontic care.

## Supplementary Material

cjag065_Supplementary_Data

## Data Availability

The data underlying this article will be shared on reasonable request to the corresponding author.
